# Case of Injury of the Leg Resulting in Mortification and Death

**Published:** 1845-10

**Authors:** Jesse Harvey

**Affiliations:** Harveysburg, Ohio


Art. VI.—*Case of Injury of the Leg resulting in Mortification and Death.* By Jesse Harvey, M. D., of Harveysburg, Ohio.

A. M., aged 20 years, a healthy young man, was jumping with some others, about four months ago, when he stepped into a hole in the ground and twisted his ankle. He felt at the time not only severe pain in the ankle, but also a sharp pain on the outside of his leg, about four inches below the knee joint. This put an end to his exercises, but he continued to walk, although he experienced some pain at the ankle as well as below the knee. Once, in the course of the three succeeding months, the leg swelled below the knee and laid him by for a few days, when he was able to go again.

About five weeks ago it began to swell, and to pain him much more than it had ever done, both at the ankle and below the knee; it was bandaged and thought to be relieved, and he continued going on it two weeks longer; the bandage failed, however, of producing the relief it had done, and the swelling below the knee increased rapidly and extended downwards.

At the first visit, 22d of June, I pronounced it a case of fracture of the fibula, a few inches below the upper extremity, and consultation, on the 23d with Drs. Fisher and Corlis, confirmed my diagnosis.

The general system by this time had suffered considerably, and with some trifling hope, but at the same time fearing the worst, we determined to try the effect of a few emetics, in case he should bear them, to produce if possible absorption; but we found the stomach much more irritable than was anticipated, and that the emetics could not be borne. We then commenced an alterative course as follows: ℞ calomel grs. iv., ipecac. grs. ii., opium gr. $\frac{1}{2}$, one to be taken every four hours until a manifest improvement was made.

This course was continued until the 30th, when he appeared to be sinking, having no desire for food of any kind. Wine was allowed, and next day he felt some desire for food; tongue very red; pulse about 100, as it had been for several days, and small. At the second consultation with Doctor Corlis, the wine was discontinued, and an incision was made into the tumor, as it appeared evident on percussion that it was filled with pus. Very little of this however was discharged; cold poultices were applied, and powders of calomel, ipecac., morphia, and camphor were continued.

July 3d—10 o'clock A. M. Evident signs of mortification. Drs. Fisher and Corlis called at 3 o'clock P. M.; pulse 150, leg numb and cold. He died at 3 o'clock on the morning of the 4th, and the tumor was dissected twelve hours after by consent of friends.

Amputation at the first consultation might have saved this patient; but this is doubtful; had the wine been continued, with barks, and the tumor not opened, it is possible he might have survived a few days longer.

August 30th, 1845.

